# Ageism, human rights and ethical aspects of end-of-life care for older people with serious mental illness

**DOI:** 10.3389/fpsyt.2022.906873

**Published:** 2022-07-28

**Authors:** Carla Kotzé, Johannes Lodewikus Roos

**Affiliations:** Department of Psychiatry, Faculty of Health Sciences, School of Medicine, Weskoppies Psychiatric Hospital, University of Pretoria, Pretoria, South Africa

**Keywords:** ageism, human rights, dignity, ethics, end-of-life, elderly, serious mental illness

## Abstract

There are many complex concepts to consider during end-of-life discussions and advance care planning, especially when vulnerable populations such as older individuals with serious mental illness are involved. This article aims to summarize some of these important concepts, such as the effects of ageism, preservation of human rights and dignity, supported or shared decision making and palliative approaches. It emerged from a study that found two thirds of 100 participants 60 years of age and older with serious mental illness had end-of-life decision-making capacity. This finding highlighted the individual and contextual nature of decision-making capacity, the importance of consideration of individual values and protection of human dignity during end-of-life care. Healthcare providers have a duty to initiate end-of-life and advance care discussions, to optimize decision-making capacity, and to protect autonomous decision-making. Chronological age or diagnostic categories should never be used as reasons for discrimination and all patients should receive end-of-life care in keeping with their preferences and values.

## Introduction

It is seen as a social obligation to provide end-of-life care with a focus on preservation of human dignity to all. During this process there are many ethical aspects that have to be considered. Consideration of the individual's values is crucial to assure autonomous decision making in any process of end-of-life or advance healthcare planning (1). The novel coronavirus disease 2019 (COVID-19) pandemic has brought to the fore many ethical questions about rationing of limited healthcare resources. Implementation of critical care triage guidelines might discriminate against older adults and individuals with cognitive or physical impairments (2). This is especially concerning when ~15% of the global population of adults 60 years of age and older have a mental disorder (3). Premature mortality and poor physical health- and end-of-life care are some of the multiple disadvantages that people with mental illness have to face (4). This highlights the need to discuss end-of-life care with patients and their families to ensure that patient preferences are incorporated into advance care planning and that patients receive care that aligns with their values (5).

Decision-making capacity can be impaired by any diagnosis or treatment that can influence mental activity and cognition (6). Most diagnoses are associated with a range of severity, and in a conscious patient, no specific diagnosis is uniformly predictive of incapacity. The contextual and specific nature of decision-making and informed consent should always be taken into account (1). When a person presents with cognitive impairment from any cause (e.g., mental illness, dementia, or delirium), the determination of decision-making capacity is critical for a proper balance to be achieved between maintaining respect for autonomy and acting in the patient's best interest (7).

## Background

A descriptive, cross-sectional, observational study was conducted at Weskoppies Hospital, Gauteng Province, South Africa. This study focused on the decision-making capacity and healthcare-related values of older people with serious mental illness. The main finding of this study was that out of 100 participants older than 60 years of age diagnosed with serious mental illness (including schizophrenia, major depressive disorder and bipolar disorder), two thirds had decision-making capacity. These patients could engage in end-of-life discussions and convey their preferences and values. During this research many ethical aspects around end-of-life care and decision-making capacity were encountered. Finding that 65% of the participants could make end-of-life decisions and discussing their values and preferences drew attention to the issues of ageism, preservation of human rights and dignity and other important ethical considerations in this particularly vulnerable population (8).

## Discussion

### Ageism

The topic of ageism received a lot of attention since the onset of the pandemic. It is a social construct defined as prejudice, discriminatory practices and stereotypes toward people because of their age including situations that compromises an older persons' human rights. This discrimination may be worsened when the older person also has a mental health problem, but this population has been largely ignored in human rights frameworks (3, 9). Elder abuse can be one of the devastating consequences of ageist attitudes. This emphasizes the need for a non-judgmental approach in geriatric mental healthcare, with a focus on safety and social justice (10). The importance of these aspects have led to issuing of a joint statement by the International Psychogeriatric Association and the World Psychiatric Association-Section of Old Age Psychiatry on the rights of older persons with mental health conditions and psychosocial disabilities to address ageism and support human rights in mental health care. This statement includes support for the United Nations Decade of Healthy Aging and convention on the rights of older persons where the focus is on safeguarding and prevention of discrimination against older people with mental illness (11, 12). Preventative measures to address ageism and legal guidance for governments to ensure the realization of rights of older people are urgently needed (9).

### Human rights and dignity

At the heart of human rights it the concept of dignity (3). Human dignity is a very nuanced concept that remains a topic of debate in bioethics. It refers to the objective value that is recognized and inherent in humans. Other conceptualisations of dignity are as a subjective self-value, as a behavioral manifestation, or as an aspirational component, but it has different meanings for different people and there is a lack of literature that focuses on dignity (13). Dignity can include, but is not limited to, factors such as autonomy, social inclusion, justice, respect, independence and privacy (3). The concept of dignity is not often included as a core goal of health systems. Certain policies implemented during the COVID-19 pandemic, such as restrictions of visits by loved ones when people are seriously ill or for people with disabilities shows the tenuous grasp of dignity as a core aspiration of health systems. The importance of dignity and protection of human rights as core principles in health care should be reaffirmed. A holistic approach that takes into consideration the social determinants of health and incorporates rights frameworks in health care is essential (14). To address all potential sources of suffering in terminally ill patients require consideration of individual, interpersonal, societal and existential factors in addition to the medical factors. Adequate resources and access to psychiatric and palliative care are required to alleviate suffering and safeguard human dignity (4, 15).

### Guiding ethical principles for end-of-life care

Healthcare professionals should be knowledgeable about ethical guiding principles pertinent to end-of-life care. The ultimate goal of determining decision-making capacity is to maintain this balance between respect for patient autonomy and protecting those who lack capacity from making harmful decisions (6, 16). The most common framework that is used for ethical reasoning in health care is principlism, with the four guiding principles of respect for autonomy, non-maleficence, beneficence, and justice. These can, at times, be insufficient to guide end-of-life care decisions, especially when two of the principles seem at odds. When dealing with morally complex situations, it has been suggested that clinicians should consider multiple ethical frameworks to find the right course of action and to prevent moral distress. During these deliberations all relevant medical issues and moral values should be addressed (16, 17).

#### Respect for autonomy

An important consideration is the link between medical ethics and African philosophy. A concern about the theory of principlism and autonomy specifically, lies in the presumption of the supremacy of the individual and a failure to acknowledge the communal nature of people. The approach to medical ethics in the African context ought to be established upon community-orientated values with complementary use of African philosophy and medical ethics (18). Keeping this in mind, clinicians still have the obligation to obtain valid informed consent to protect an individual's right to self-determination. This requires a competent person to make a voluntary choice after the disclosure of appropriate and sufficient information, in the absence of undue influence (6). The use of advance healthcare directives to preserve patient autonomy at times of incapacity, and effective communication about end-of-life decisions between patients, families and any other relevant parties should be encouraged (19).

The preferences of all patients, including older patients with mental illness or cognitive impairment should be respected in all areas where the illness is not affecting their decision making. This includes their right to refuse treatment, even if the refusal may result in their death. The decision-making process can be very complex and the limitations to autonomy should always be taken into consideration. Doctors cannot behave in unethical ways because of requests from patients, and other ethical principles might eclipse autonomy in the process of choosing the most ethical course of action (20).

#### Beneficence/non-maleficence

Even while respecting autonomy, one should be aware of its limitations to enable provision of care that benefit the patients, while also preventing harm. When considering the principles of beneficence and non-maleficence, potential benefits should be considered and balanced with the requirement not to cause harm. Patients should understand that they can refuse any treatment, and the potential side effects or risks should be made clear, especially when the potential benefit might be limited. Explanations should be on the patient's level of knowledge and follow-up discussions might be required for clarification on more complex issues (21). The goals of treatment are always important considerations and should be reassessed when there is a change in the patient's clinical condition. In situations where one has to decide if treatment should be withheld or withdrawn, the context, benefits, and potential harm should always be considered. These decisions must be guided by the wishes of patients and their families as well as the medical team (22).

#### Justice

Justice is the principle that refers to fairness and equitable distribution of resources, with special protection for vulnerable groups or people with substantial impairment in their ability to protect their own interest (21). The kind of justice of importance in the public health sector is distributive justice and is critical in decisions about health resource allocation. The COVID-19 pandemic overwhelmed healthcare systems by the increased demand for care, but it still remains important to apply any guidelines on resource allocation fairly and consistently to all patients (23).

#### Other ethical considerations

The doctrine of double effect is grounded in the ethical principle of proportionality and is often cited when palliative sedation is used. In palliative sedation, medication is used to relieve intolerable symptoms (desired good outcome), but may cause loss of function or hasten death. This possible predictable outcome will not be intentional, thereby not considered ethically problematic. Symptom relief without killing the patient should be an achievable and realistic goal and it should be a benefit that is proportional to potential negative consequences. Advances in medicine has made effective symptom control without endangering the patient's life more achievable (24).

In the utilitarian or consequentialist view, the balance of benefits and burdens should be maximized. An example of this approach would be when the decision to use resuscitation has to be made. In each specific case, one would weigh the likelihood of survival and subsequent quality and duration of life against potential suffering and costs. A common scenario in end-of-life care where the deontological view is used is when clinicians argue for withdrawal of futile treatment based on a utilitarian view, but family members request continued treatment out of a sense of duty. With the deontological view, duties transcend the calculation of benefit. The communitarian view emphasizes communal values, social goals, cooperation, and the common good to improve quality of life for the entire community. With virtue ethics, the moral character that informs behavior will be the focus. In end-of-life care, virtues will include advocacy, compassion, and justice. These frameworks are alternative considerations for those ethically challenging situations where principlism principles are at odds (16).

### Supported and shared decision-making

Supported decision-making is used to empower individuals to make their own decisions to the maximum extent possible. This approach increases self-determination and should be implemented before resorting to substitute decision-making (25). More research is needed in this field, but there are important arguments such as human rights, effectiveness and pragmatism that support this approach in mental health care (26). An alternative to this approach can be shared decision-making where patients and healthcare providers make decisions in a partnership. In these scenarios it will be important to eliminate power asymmetries, to consider patients values and preferences in combination with the best medical evidence. In all scenarios, except requests for futile treatment options or requests that will not be considered legal, the patients' choice should be respected even if it is different from the healthcare providers recommendations (27, 28). There is still a lack of implementation of shared decision-making, but research has shown that most people with mental health problems are willing to engage in this approach. Conveying information in an easily accessible way with mutual respect and trust are essential components of this process (29).

### Palliative care

The integration of palliative care services into mental healthcare is considered to be long overdue (4). A palliative approach can promote equitable health services and access to palliative care is considered a human right (30). With this approach dying is considered a normal process, but the World Health Organization (WHO) has estimated that only 14% of people receive palliative care when it is needed (31, 32). There is consistent evidence that individuals with serious mental illness such as schizophrenia experience disparities in care such as lack of access to quality end-of-life care contributing to poorer outcomes. This highlights the importance of a multidisciplinary team approach to protect patients with serious mental illness from harm through stigmatization, possible incapacity to make decisions, inability to engage in treatment or maintain adequate self-care, and poor understanding of theirs illness (33, 34).

Palliative principles like focusing on quality of life, effective communication about goals of care, reducing symptom burden and advance care planning should be implemented. Instead of focusing only on life-limiting illness, relief of suffering should be emphasized and implementation of this approach for patients with serious mental illness should be an early consideration (32, 33). When a palliative approach is applied in psychiatry it shifts the focus form a curative approach to an approach that promotes person centredness and quality of life (35). Psychiatric services should integrate palliative care into programs when caring for patients with serious mental illness, especially during this time when the ongoing pandemic and climate change poses significant threats to the well-being of older people. This will ensure that the right to dignity, autonomy, and self-determination will be respected and individualized care will be provided in keeping with the values of even the most vulnerable person (36–40).

## Clinical care, research and policy implications

Caring for elderly patients with mental illness can pose many challenges and this can be exacerbated by a life-threatening medical condition. To be able to provide optimal care for all patients with serious mental illness healthcare providers should be trained in individualized, evidence-based approaches to decision-making capacity assessments. It should be done in a quiet environment, when a patient is comfortable and rested. Time pressure and undue influence should be eliminated, while encouragement and shared or supported decision-making are considered essential. Training in and implementation of these measures to enhance autonomous decision-making can ultimately reduce the stigma associated with serious mental illness in older populations (41, 42).

The stability of healthcare preferences over time in older patients with serious mental illness is an area that has not received frequent attention and warrants further investigation including qualitative approaches to gain a more in-depth understanding of their values and personal preferences. Topics that may be explored include the preferred place of death, preferences for avoiding hospitalization or implementation of do-not-resuscitate orders, and how these are influenced by personal and cultural values and psychiatric symptoms.

Policies in mental healthcare facilities should address issues around end-of-life care and should make provision for routine implementation of advance care planning. This should be implemented before urgent care is required and at times when psychiatric symptoms is sufficiently controlled. This will ensure that even when acute illness impairs a patient's ability to give informed consent, the care provided will still be in keeping with their preferences. These discussions should be documented in as much detail as possible on a platform that is accessible by all healthcare providers that might be involved with a patient's care and it should be updated regularly. Attention should be given to preferences about place of care, level of care, and goals of care, such as prolonged survival, optimisation of functioning or comfort, achieving life goals, and the support for family or caregivers. It might be useful to have any advance care discussions that culminate in specific treatment orders, such as do-not-resuscitate orders, written in a standardized format that is easy to access and understand during emergencies (43, 44). This will not only ensure optimal and preferred care at times of incapacity, but can also protect medical practitioners from potentially difficult medico-legal consequences and moral distress when they have to make difficult decisions about resource allocation and appropriate levels of care.

## Conclusion

Regardless of the decision-making capacity of a patient, transition to palliative care should always be an early consideration for any patient with chronic conditions, especially if these conditions are considered life-limiting. The palliative approach can be valuable in patients with chronic, treatment-resistant serious mental illness even without comorbid medical problems. In a truly palliative approach, there should be an awareness of the limitations associated with the prognosis. Integration of psychiatric care and palliative care should be a priority for all facilities where long-term care for those with serious mental illness are provided (37, 38, 45–47). Balancing of rights and ethical principles are important considerations in the provision of end- of-life care. In older adults with serious mental illness supported or shared decision-making, advance care planning and early transition to a palliative approach are encouraged. Mental health professionals have a duty to approach this vulnerable group with empathy, without judgment and to provide holistic care that protects human rights and preserves dignity.

## Data availability statement

The original contributions presented in the study are included in the article/supplementary material, further inquiries can be directed to the corresponding author/s.

## Ethics statement

The studies involving human participants were reviewed and approved by University of Pretoria's Faculty of Health Sciences Research Ethics Committee. The patients/participants provided their written informed consent to participate in this study.

## Author contributions

CK and LR were involved with the original research that this perspective grew from. All authors contributed to the writing of this perspective article.

## Conflict of interest

The authors declare that the research was conducted in the absence of any commercial or financial relationships that could be construed as a potential conflict of interest.

## Publisher's note

All claims expressed in this article are solely those of the authors and do not necessarily represent those of their affiliated organizations, or those of the publisher, the editors and the reviewers. Any product that may be evaluated in this article, or claim that may be made by its manufacturer, is not guaranteed or endorsed by the publisher.
